# The change-makers of West Africa

**DOI:** 10.1186/s12961-017-0208-6

**Published:** 2017-07-12

**Authors:** Sue Godt, Sharmila Mhatre, Anne-Marie Schryer-Roy

**Affiliations:** 1 Maternal and Child Health Program, International Development Research Centre, PO Box 62084, 00200 Nairobi, Kenya; 2 Open Society Foundation, New York, NY 10019 United States of America; 3 Independent Consultant, PO Box 91, 00606 Nairobi, Kenya

## Abstract

West Africa was the focus of global attention during the Ebola virus disease outbreak, when systemic health system weaknesses compounded a serious emergency and complicated response efforts. Following the crisis, calls were made to strengthen health systems, but investments to date have fallen short of delivering the support needed to build strong health systems able to prevent and manage future outbreaks.

In part, this reality serves to highlight the shortcomings of the solutions being repeatedly prioritised by external funders and experts, solutions that often fail to consider the wealth of West African evidence and actors actively working to strengthen the leadership and health systems needed to drive and sustainably improve national health outcomes. Unfortunately, this knowledge and experience are rarely heard in the global arena.

This journal supplement is a contribution, although small, to changing this practice by putting the perspectives, experiences and knowledge of West Africans on the table. It presents findings from a series of research and capacity development projects in West Africa funded by the International Development Research Centre's Maternal and Child Health programme (formerly Governance for Equity in Health Systems).

The evidence presented here centres around two key themes. First, the theme that context matters. The evidence shows how context can change the shape of externally imposed interventions or policies resulting in unintended outcomes. At the same time, it highlights evidence showing how innovative local actors are developing their own approaches, usually low-cost and embedded in the context, to bring about change. Second, the collection of articles discusses the critical need to overcome the existing fragmentation of expertise, knowledge and actors, and to build strong working relationships amongst all actors so they can effectively work together to identify priority issues that can realistically be addressed given the available windows of opportunity.

Vibrant West African-led collaborations amongst researchers, decision-makers and civil society, which are effectively supported by national, regional and global funding, need to foster, strengthen and use locally-generated evidence to ensure that efforts to strengthen health systems and improve regional health outcomes are successful. The solutions are clearly not to be found in the ‘travelling models’ of standardised interventions.

## Introduction

West Africa was the focus of global attention during the Ebola virus disease outbreak [1, 2]. The epidemic dramatically demonstrated how systemic health system weaknesses, including weak or non-existent national disease surveillance systems, compounded a serious emergency and complicated response efforts [3–6]. Although calls were made to strengthen health systems as part of post-Ebola reconstruction efforts, investments to date have fallen short of delivering the support needed to build strong health systems able to prevent and manage future outbreaks [7].

In part, this shortcoming is due to the challenge that health system strengthening poses. However, at the same time, it also highlights the failings of the solutions being repeatedly prioritised by external funders and experts. The missing headline in the narrative surrounding the Ebola virus epidemic is that West Africa is home to a wealth of evidence, researchers, practitioners, policymakers and civil society actors actively working to strengthen the leadership and health systems needed to drive and sustainably improve national health outcomes. Unfortunately, this knowledge and experience are rarely heard in the global arena. The research and development aid agendas, discourse and investments tend to be driven by external voices, interests and funds, with little space for West African leaders and researchers to share their insights and experiences. This journal supplement is a contribution, however small, to changing this practice by putting the perspectives, experiences and knowledge of West Africans on the table. It aims to be reflective, and looks at how the array of stakeholders – from local to global – can more effectively play their part to support health systems strengthening efforts in West Africa.

In 2011, the International Development Research Centre’s (IDRC) Governance for Equity in Health Systems programme, now named the Maternal and Child Health programme, embarked on a concerted effort to strengthen research in West Africa to improve equitable health systems. Systematic consultations with stakeholders in the region pointed to persistent challenges that negatively impacted on health. Health systems failed to promote good health and provide quality and sustainable services for the most vulnerable. The research environment did not enable the building of relevant skills or catalyse the needed resources to mount comprehensive research programmes that would strengthen health systems and address national priorities. Researchers were often fragmented by discipline, language and national boundaries. Even with relevant findings, there were weak research-to-policy-and-practice mechanisms and processes to apply the results.

Working with the West African Health Organisation (WAHO), a regional body with the mandate to engage with the 15 member states of the Economic Community of West African States (ECOWAS) to strengthen policy and collective practice to improve health outcomes, a plan was developed that prioritised overcoming this fragmentation. These issues were subsequently integrated into a regional call for concept notes. Based on the recognition that change cannot be imposed from the outside but must be driven from within the region, the main objective was to strengthen a critical mass of researchers, research institutions, practitioners and decision-makers to undertake and apply relevant research to strengthen health systems and contribute to improving health outcomes. This journal supplement is one of the outputs of IDRC’s programme of work to strengthen the research environment.

## Emerging themes

The articles in this supplement present evidence highlighting the barriers to sustainable innovation and change. They also demonstrate why health systems need to be holistically strengthened given how vertical interventions focusing on one specific outcome can have skewing effects on the delivery and impact of related healthcare services [8–11]. Context also matters – it is not enough to simply ‘adapt’ promising innovations developed elsewhere. The evidence shows how context can change the shape of externally imposed interventions or policies resulting in unintended outcomes [12]. Evidence is brought forward by Defor et al. [13] on the extent of the geographical and linguistic divide in the production of research. This fragmentation exacerbates efforts to undertake the most needed research to address health challenges.

At the same time, the articles demonstrate how efforts are underway to strengthen the environment for generating and using evidence to support sustainable change [14, 15]. To mitigate some of the fragmentation of geography, discipline and language, WAHO demonstrates how governance of research, combined with strong policy and practice engagement platforms, can play a supportive role [16, 17]. This foundation is helping to strengthen research and stakeholder collaborations at national and regional levels.

### Context matters

A key theme emerging from the articles underlines the importance of the scope of interventions as well as the context in which these are developed. In Sierra Leone, Koroma et al. [8] examine efforts to provide free maternal healthcare in the rural Bombali district prior to the Ebola virus disease outbreak and find that, despite high demand by the population, poor quality of services undermined results. Poor infrastructure, inadequate skilled staff and low availability of supplies, combined with structural inequities and lack of sustainable funding mechanisms, compromised service delivery. In Burkina Faso, Yaogo [11] draws on previous research into policies to abolish – in part or in full – various health service fees and identifies similar constraints to providing accessible quality services. Both articles note the skewed impact of externally funded programmes. In Sierra Leone, being part of a national donor-driven programme resulted in a much higher rate of testing pregnant women for HIV than for other antenatal tests [8]. In Burkina Faso, there were challenges in managing the numerous different, often once-off, donor contributions to subsidise disease prevention and treatment [11].

Duclos et al. [10] examine mHealth interventions in the Nouna health district in Burkina Faso from the perspective and expectations of front-line health workers and users. Although useful for building a support network, mobile phones could not address key underlying barriers to accessing antenatal care, including poverty, gender issues and geographical distances. The authors caution against applying universalistic approaches to mHealth and advocate for “*careful project design and policymaking attentive to the knowledges and practices of local communities*”.

Using maternal newborn and child health as an example, Agyepong et al. [18] highlight how context along with health system factors (the WHO’s foundational building blocks combined with people, power, processes and values) have played either enabling or limiting roles to undermine or enhance the impact of health interventions. As the authors note, numerous ‘proven’ or ‘presumed’ effective interventions to address challenges such as maternal and child mortality have been implemented, but outcomes have not always improved as expected.

Olivier de Sardan et al. [12] are developing a grounded theory to “*understand the relationship between standardised interventions and implementation contexts, and the many unexpected, invisible or perverse effects*” that result. The authors explore in detail the ‘revenge of the context’, and through an analysis of the development and dissemination of ‘travelling models’ of standardised interventions, they point to an evidence base in Niger and other countries showing the disconnect between externally-driven interventions and local conditions, and social and professional norms and standards. International institutions, non-governmental organisations and other actors often play the role of ‘travel agencies’ facilitating the transfer of such models. The authors conclude by pointing to evidence showing how innovative local actors are developing their own approaches, usually low-cost and embedded in the context, to bring about change.

### Overcoming the fragmentation

As a second theme, several authors identify the critical need to overcome the existing fragmentation of expertise, knowledge and actors by strengthening collaboration across expert institutions and individuals. They also point to the need to build strong working relationships amongst researchers, decision-makers and practitioners, so they can effectively work together to identify priority issues that can realistically be addressed given the available windows of opportunity.

Sombie, Aidam and Montorzi [16] discuss how national health research systems in four fragile states were supported to build the foundation for undertaking and using relevant health research. Despite contextual challenges, such as political instability and the outbreak of the Ebola virus, some progress was made towards improved national governance by developing national health research policies and priorities, strengthening ethics review and building a regional research information system. The authors reflected on the strategic accompaniment role played by WAHO as a regional organisation and on the need for international players to support advocacy, resource mobilisation and capacity strengthening activities that respond to national and regional priorities.

Studying the health policy and systems research literature, Defor et al. [13] describe the patterns and trends of Anglophone and Francophone peer-reviewed publications across ECOWAS countries from 1990 to 2015. Although their findings show increased research production rates since 2008, the region still lags far behind other regions. Nigeria, Burkina Faso and Ghana account for over 70% of the publications, most of which are written in English. Given that health policy and systems research is context specific, the authors point to an urgent need for “*local actors with an understanding and appreciation of their own health systems challenges to drive the processes of evidence generation and application*” [13]. The authors argue for greater cross-country institutional collaboration that emphasises joint research agenda setting with research consumers as one way of overcoming the current fragmentation.

As mechanisms for influencing policy and practice vary and are weak in many countries, Keita et al. [15] report on a regional initiative aimed at stimulating, promoting and strengthening collaboration across researchers, actors and decision-makers to undertake and use research results to improve health systems governance and equity. Based on evidence in the literature, WAHO encouraged the establishment of steering committees to accompany four health systems research projects in Burkina Faso, Nigeria, Senegal and Sierra Leone. Each committee took a different form depending on local and national contexts, and this flexibility strengthened implementation. In rural Sierra Leone, for example, the local steering committee included community and district stakeholders from transportation, defence and security sectors who were all committed to making the free maternal health policy work for everyone. This experience has contributed to developing longer-term relationships amongst research teams and decision-makers. Nevertheless, and given the dependence on the funded researchers for resources, the need for autonomy and strengthened agency of all committees was identified.

Uneke et al. [14] explore the deepening demand for evidence through assessing Nigerian policymaker and stakeholder perceptions about their needs for, as well as barriers and facilitators to, using research evidence in policymaking. Identified barriers include “*inadequate capacity of organisations to conduct policy-relevant research, inadequate budgetary allocation for policy-relevant research, policymakers’ indifference to research evidence, poor dissemination of research evidence to policymakers and lack of an interaction forum between researchers and policymakers*” [14]. The study highlights the need to strengthen individual and institutional capacity as well as improve research infrastructure and funding. Importantly, the assessment also identifies the need to establish sustainable platforms for policymaker–researcher interaction.

## Concluding comments

The articles in this supplement give pause for reflection on the governance of health research, on whose agendas matter, and on the importance of starting implementation from the local level to inform global arenas. At national levels, governments make commitments to strengthening the health sector [19] and health research [20], but often do not meet their obligations. The situation is confounded by the tensions surrounding funder agendas, priorities and practices, and their impact in the region, in particular the skewing effects of vertical programmes.

Despite good intentions, there is clearly a mismatch between the intersection of global, regional and national governance in West Africa. The complex history of the region, with its colonial and linguistic divides, continues to influence institutions, norms and practice. Vibrant West African-led collaborations amongst researchers, decision-makers and civil society, which are effectively supported by national, regional and global funding, need to foster, strengthen and use locally-generated evidence to ensure that efforts to strengthen health systems and improve regional health outcomes are successful. The solutions are clearly not to be found in the ‘travelling models’ of standardised interventions.

Such a developmental approach was reflected in the Millennium Development Goal era commitments made in the Paris Declaration on Aid Effectiveness and the Accra Agenda for Action, which prioritised harmonisation of development assistance and alignment with regional and national priorities. While that vision was not fully realised [21], these issues are once again under discussion in the context of achieving the Sustainable Development Goals and in calls for development of a Framework Convention on Global Health [22].

There is no easy answer, but we hope that this supplement will contribute to an ongoing dialogue about these important issues.

## Abbreviations

ECOWAS: Economic Community of West African States
IDRC: International Development Research Centre
WAHO: West African Health Organisation
